# Catalytic Efficiency Improvement in Cellobiohydrolase I by Cross-Species Domain Exchange Engineering

**DOI:** 10.3390/ijms26094024

**Published:** 2025-04-24

**Authors:** Jing Xue, Xianzhang Jiang, Anjing Li, Jiaxin Li, Xiaoyun Su, Jianzhong Huang, Lina Qin

**Affiliations:** 1 National Joint Engineering Research Center of Industrial Microbiology and Fermentation Technology, College of Life Sciences, Fujian Normal University, Fuzhou 350117, China; jingxue07@126.com (J.X.); jiangxz@fjnu.edu.cn (X.J.); lianjing6521@163.com (A.L.); 2 State Key Laboratory of Animal Nutrition and Feeding, Institute of Animal Sciences, Chinese Academy of Agricultural Sciences, Beijing 100193, China; jessielee6630@163.com (J.L.); suxiaoyun@caas.cn (X.S.)

**Keywords:** Cellobiohydrolase I (CBHI), cellulose, carbohydrate-binding module (CBM), *Trichoderma reesei*, *Chaetomium thermophilum*

## Abstract

Understanding the molecular mechanisms of cellobiohydrolase I (CBHI), a key enzyme in cellulase complexes, is crucial for developing efficient enzymes for the degradation of lignocellulosic biomasses (LCB). Building on our previous discovery that *Chaetomium thermophilum* CBHI (C-CBH) exhibits significantly higher specific activity than *Trichoderma reesei* CBHI (T-CBH), systematic domain-swapping experiments were conducted to elucidate the structural determinants of catalytic efficiency in CBHI. Herein, the carbohydrate-binding modules (CBM) of the CBHIs from *Trichoderma reesei* (T-CBH) and *Chaetomium thermophilum* (C-CBH) were interchanged and to obtain two chimeric mutants TC-CBH and CT-CBH. These four CBHs were expressed in *T. reesei*, and the enzyme properties were analyzed. Comparative characterization revealed that while module exchange preserved native temperature/pH adaptability, it significantly altered substrate specificity and catalytic performance. The CT-CBH variant was identified as the most efficient biocatalyst, exhibiting four key advantages over T-CBH: (1) protein expression levels that far exceed those of T-CBH, (2) specific activity enhanced by 2.6-fold (734.5 U/μM vs. 282.5 U/μM on MU-cellobiose), (3) superior degradation capacities for filter paper (1.6-fold) and xylan, and (4) improved binding affinity for crystalline cellulose. These findings establish cross-species domain engineering as a viable strategy for creating high-performance cellulases, providing both mechanistic insights and practical solutions for lignocellulose degradation.

## 1. Introduction

Lignocellulosic biomass (LCB), the most abundant renewable carbon source on Earth with an annual yield of 1.5–1.7 × 10^11^ tons, represents a promising feedstock for sustainable biofuel production [1,2]. However, the crystalline structure of cellulose, its primary component composed of β-1,4-linked glucopyranoside chains, poses significant challenges to enzymatic hydrolysis, thereby necessitating the development of optimized cellulases for efficient industrial saccharification [3,4]. Among cellulolytic enzymes, cellobiohydrolase I (CBHI) is particularly critical due to its ability to processively cleave crystalline cellulose into cellobiose [5]. Despite constituting over 60% of the secreted cellulases in *Trichoderma reesei*, the industrial utility of CBHI is constrained by its relatively low specific activity, highlighting the need for improved variants to enhance LCB conversion efficiency [6].

Significant progress has been made in elucidating the degradation mechanisms of CBHI through structural and functional analyses, advancing the understanding of its role in LCB conversion. Research has demonstrated that CBHI preferentially binds to amorphous cellulose regions where lignin is absent, while high lignin content in adjacent areas can inhibit enzyme adsorption [7]. This observation led to the identification of a tyrosine residue implicated in the non-productive adsorption of CBHI onto lignin, highlighting a key factor in enzyme-substrate interactions [8]. Enhancing thermostability has emerged as a critical strategy to improve CBHI’s catalytic efficiency by introducing disulfide bonds and incorporating proline residues to augment protein stability at elevated temperatures [9,10,11]. Additionally, domain-swapping experiments have revealed the potential of chimeric enzyme engineering. For instance, the catalytic domain of *Talaromyces emersonii* was successfully fused with the linker and cellulose-binding module (CBM) of *T. reesei* CBHI, generating a chimeric *TeTr*CBH I enzyme. This construct demonstrated enhanced hydrolytic performance, underscoring the importance of inter-domain synergy in catalysis [12,13,14]. Notably, domain exchange experiments attributed the superior hydrolytic efficiency of *T. reesei* CBHI to specific structural features of its linker and CBM, further emphasizing the role of these regions in substrate recognition and processivity [15]. These advancements collectively provide a foundation for rational enzyme engineering to optimize CBHI for industrial LCB saccharification applications.

Our laboratory previously demonstrated that cellulases secreted by *Chaetomium thermophilum* exhibit superior specific activity, thermostability, and stress tolerance compared to those of *T. reesei* [6]. Notably, the specific activity of *C. thermophilum* CBHI was significantly higher than that of *T. reesei* CBHI, despite both enzymes sharing a conserved modular architecture comprising a catalytic domain (CD), linker peptide, and carbohydrate-binding module (CBM). This observation raises a critical question: how do these structural domains contribute to the enhanced cellulolytic efficiency of *C. thermophilum* CBHI? Previous studies have shown that the CBM of *T. reesei* CBHI plays a pivotal role in crystalline cellulose degradation, outperforming homologous CBMs from other fungi such as *Penicillium oxalicum* [15]. To investigate the structural basis of this difference, domain-swapping experiments were conducted between *T. reesei* and *C. thermophilum* CBHI, generating chimeric mutants with varying combinations of CDs, linkers, and CBMs. Recombinant CBHI variants were expressed in *T. reesei*, and their biochemical properties were systematically compared with the wild-type enzymes. This study revealed that the CBM significantly influences the catalytic behavior of the CD, thereby affecting overall cellulolytic efficiency. These findings highlight the CBM as a key determinant of cellulase performance and underscore its potential applications in enzyme engineering, including its use as a purification tag or for cellulose fiber modification [16].

## 2. Results and Discussion

### 2.1. Recombinant Expression of Two CBHIs and Their Chimeric Mutants

**Figure 1:**
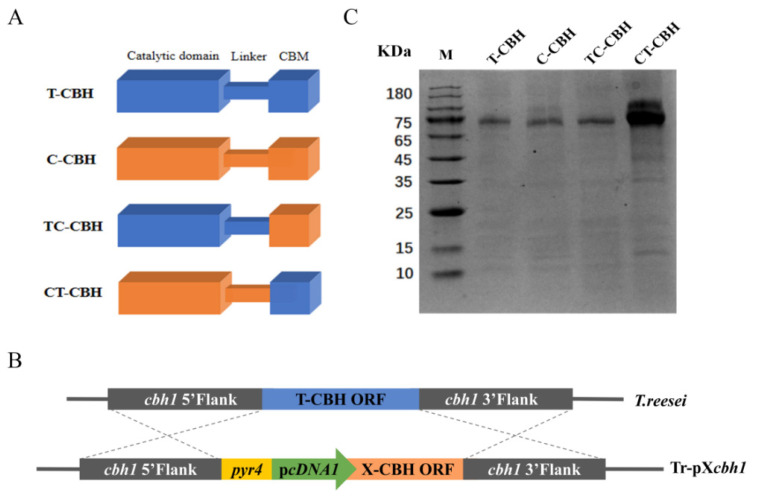
Recombinant expression of CBHI wild-type and two chimeric mutants. (**A**) Schematic diagram of CBHI domain swapping. (**B**) Schematic diagram of the construction of the recombinant strains Tr-pT*cbh1*, Tr-pC*cbh1*, Tr-pTC*cbh1*, and Tr-pCT*cbh1*. X represents T, C, TC, and CT; Tr-pX*cbh1* represents Tr-pT*cbh1*, Tr-pC*cbh1*, Tr-pTC*cbh1*, and Tr-pCT*cbh1*, respectively. (**C**) Expression of CBHI composed of four different structural domains in *T. reesei*. T-CBH: *T. reesei* CBHI; C-CBH: *C. thermophilum* CBHI; TC-CBH: chimeric construct with the CBM of *T. reesei* CBHI changed to that of *C. thermophilum*; CT-CBH: chimeric construct with the CBM of *C. thermophilum* CBHI replaced by that of *T. reesei*.

*T. reesei* is able to secrete a variety of cellulases from different glycoside hydrolase (GH) families, with CBH I accounting for up to 60% of the overall extracellular proteins [17]. This suggests that CBHI must play a critical role in cellulose degradation. Similar to many other GHs, CBHI consists of tandemly linked catalytic domain (CD), linker, and CBM. Although the functions of these domains have been defined, there are reports suggesting that a domain may have more intrinsic effects on others than previously realized. To gain more insights into the effects of the different structural domains on the function of CBHI, two CBHIs were selected from *T. reesei* (T-CBH) and *C. thermophilum* (C-CBH), respectively, for comparison. In addition, two chimeric mutants were designed by replacing the CBM of T-CBH with that of C-CBH (termed TC-CBH) or vice versa (termed CT-CBH) (Figure 1A). The plasmids containing the wild-type (pT*cbh1* and pC*cbh1*) and chimeric mutant CBHI genes (pTC*cbh1* and pCT*cbh1*) were constructed using *cDNA1* promoter and the genes were transformed and homologously integrated into the *cbh1* locus of *T. reesei* (Figure 1B). *T. reesei* cellulase secretion is glucose-retarded and cellulose-induced, and many cellulases secreted under the induced conditions have a certain degree of interference with the analysis of CBHI enzymatic properties. The *cDNA1* promoter is a constitutive promoter that allows the expression of proteins that are not carbon metabolism-retarded (CCR) under conditions in which glucose is the carbon source, whereas the secretion of other cellulases is retarded [18]. Therefore, the use of the *cDNA1* promoter facilitates the obtainment of CBHI of a relatively homogeneous composition when incubated with glucose as a carbon source. As shown in Figure 1C, all four recombinant *T. reesei* strains Tr-pT*cbh1*, Tr-pC*cbh1*, Tr-pTC*cbh1*, and Tr-pCT*cbh1* proteins could successfully express and secrete recombinant CBHI into the supernatant under glucose carbon source. Interestingly, the expression levels of CT-CBH were much higher than the rest of the others (Figure 1C). To further prove that the observed results were not by chance, nor due to experimental error, three independent repetitive transformation experiments were performed, and three independent transformants of each transformation were picked for analysis. The results were all consistent with those shown in Figure 1C. Considering that the promoter, terminator, signal peptide, and insertion site used for these recombinant CBHI gene are identical in the four different CBHI recombinant strains, this result suggested that the sequence composition of CT-CBH might be the reason for its high-level expression in *T. reesei*. However, the specific mechanisms underlying this phenomenon require further investigation.

### 2.2. The Mutant CBHIs Have Similar pH and Temperature Profiles to Their Corresponding Wild-Types

**Figure 2:**
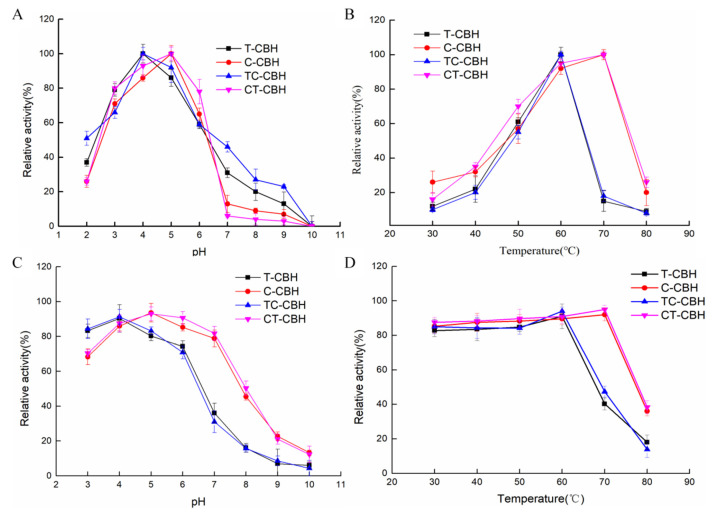
Effects of pH and temperature on the activity of the four CBHIs. (**A**) Determination of the optimum pHs of T-CBH, C-CBH, TC-CBH, and CT-CBH. (**B**) Determination of the optimum temperatures of T-CBH, C-CBH, TC-CBH, and CT-CBH. (**C**) Analysis of the pH stability of T-CBH, C-CBH, TC-CBH, and CT-CBH. (**D**) Analysis of the thermo-tolerance of T-CBH, C-CBH, TC-CBH, and CT-CBH. T-CBH: *T. reesei* CBHI; C-CBH: *C. thermophilum* CBHI; TC-CBH: chimeric construct with the CBM of *T. reesei* CBHI changed to that of *C. thermophilum*; CT-CBH: chimeric construct with the CBM of *C. thermophilum* CBHI replaced by that of *T. reesei*. The values show the mean of three biological replicates and the error bar indicates the standard deviation.

Using MUC as the substrate, each of the four purified CBHIs was incubated in a buffer at pH 2.0–10.0 for 1 h at room temperature and the optimal pHs were determined. It was demonstrated that all four CBHIs are acidic enzymes, with the optimal pHs of T-CBH and TC-CBH being 4.0 and those of C-CBH and CT-CBH being 5.0 (Figure 2A). To determine the pH stability, each of the four purified CBHIs was diluted 10-fold in 50 mM citric buffer, pH 4.8, and incubated in a buffer at pH 3.0–10.0 for 1 h at room temperature before measuring the residual activity. The results showed that T-CBH and TC-CBH were more stable at pH 3.0–5.0 by retaining more than 80% of residual activities (Figure 2C). When the pH exceeded 6.0, the enzyme activity decreased sharply and the residual enzyme activity tended to be almost zero after pH exceeded 7.0. C-CBH and CT-CBH were more stable at pH 5.0–7.0 by retaining above 80% residual enzyme activity. Correspondingly, all four enzymes were relatively stable in acidic buffers but largely decreased their activities when the pH turned alkaline. Thus, swapping the two CBMs did not affect their pH optima and tolerance.

Using MUC as the substrate, the optimum temperatures and thermal stability of the four CBHIs were investigated by incubating them in citrate buffer (50 mM, pH 4.8) for 1 h at different temperatures from 30–80 °C. While the optimum temperatures of T-CBH and TC-CBH were 60 °C, those of C-CBH and CT-CBH were 70 °C (Figure 2B). The residual enzyme activities of both C-CBH and CT-CBH at temperatures below 70 °C were above 80% (Figure 2D), indicating that they were more stable than T-CBH and TC-CBH. Therefore, the CD (and linker) must be the main factor affecting the temperature profiles of the T-CBH and C-CBH. Changing the CBM did not change the temperature optima and thermos-tolerance for T-CBH and C-CBH.

### 2.3. Kinetic Analysis of the CBHIs on MUC

The four recombinant CBHIs were individually incubated with MUC for 3 min and their specific activities were determined. The specific activities of T-CBH, C-CBH, TC-CBH, and CT-CBH were measured at 282.5 U/µM, 320.2 U/µM, 301.3 U/µM, and 734.5 U/µM, respectively (Table 1). For T-CBH, replacing the CBM resulted in a slight increase in specific activity, though not statistically significant. In contrast, C-CBH exhibited a significant enhancement in specific activity following CBM replacement. These results indicate that altering the CBM of CBHI can improve its activity on the soluble artificial substrate, MUC, although the extent of the improvement varies. C-CBH-specific activity exceeded that of T-CBH, consistent with previous findings [6]. Notably, CT-CBH demonstrated a significantly higher specific activity than the other recombinant strains, indicating its superior cellobiohydrolase activity.

The initial velocities were determined and plotted against the substrate concentrations of MUC, and the kinetic parameters of the four CBHIs were estimated by fitting the data to the Michaelis-Menten equation using a nonlinear regression method. The *K_M_* (1.3 and 1.1 mM for T-CBH and C-CBH, respectively, Table 1) and *k*_cat_ (77.5 and 93.7 min^−1^ for T-CBH and C-CBH, respectively) parameters for the two wild-type CBHIs were similar. Changing the CBM of C-CBH to obtain the chimeric mutant CT-CBH largely lowered the *K_M_* (1.1 mM to 0.7 mM), indicative of elevated affinity for MUC. However, the turnover number did not change much for this chimeric mutant from *k*_cat_ value. In contrast, changing the CBM of T-CBH to obtain the chimeric mutant TC-CBH nearly doubled the *k*_cat_, although the *K_M_* did not change. This indicated that the CBM domain has a very important but different effect on the catalytic behavior of the CBHI, even for this small and artificial substrate. The experimental results show that C-CBH has a higher conversion efficiency on MUC than T-CBH, consistent with previous studies which have reported that *C. thermophilum* often exhibits greater catalytic efficiency and affinity for their substrates compared to *T. reesei*, particularly for complex substrates like MUC [19].

### 2.4. Domain Swapping Had Different Effects on the Catalytic Performance of the CBHIs on Cellulose

Regarding the specific activity on crystalline cellulose, changing the CBM had the largest effect on the T-CBH, which was reflected by the specific activity of 3.8 µM glucose equivalents/min/µM enzyme for T-CBH on Avicel, compared to 2.0 µM glucose equivalents/min/µM enzyme for TC-CBH (Table 2). This decrease was not so obvious for the filter paper substrate, as the specific activities were 9.9 and 7.5 µM glucose equivalents/min/µM enzyme for T-CBH and TC-CBH, respectively. Replacing the CBM in C-CBH also decreased the specific activity on Avicel, albeit to a lesser extent. However, this was not observed for the filter paper substrate as the specific activities were quite comparable (10.0 and 11.7 µM glucose equivalents/min/µM enzyme for C-CBH and CT-CBH, respectively).

The apparent kinetic parameters of the CBHIs on the non-crystalline cellulose (PASC) were also compared. Changing the CBM of T-CBH simultaneously increased the *k_cat_* (2.0 × 10^−1^ to 3.2 × 10^−1^ s^−1^) and *K_M_* (5.6 × 10^−2^ to 1.2 × 10^−1^ mg/mL), with the catalytic efficiency slightly decreasing from 3.7 to 2.7 s^−1^ mL mg^−1^ (Table 2). In contrast, alternating the CBM in C-CBH had the opposite effect: the *k_cat_* decreased from 3.3 × 10^−1^ (for C-CBH) to 2.1 × 10^−1^ s^−1^ (for CT-CBH), while the *K_M_* also decreased from 7.3 × 10^−2^ mg/mL (for C-CBH) to 3.5 × 10^−2^ (for CT-CBH). However, the catalytic efficiency only slightly increased from 4.5 (for C-CBH) to 6.0 s^−1^ mL mg^−1^ (for CT-CBH). Among the recombinant CBHIs, CT-CBH showed the highest substrate affinity for PASC and the highest catalytic efficiency (*k_cat_*/*K_M_*). In conclusion, changing the CBM of CBHI affects the substrate affinity and catalytic efficiency of CBHI for PASC.

### 2.5. Hydrolysis of Different Biomass by the Four Recombinant CBHIs

**Figure 3:**
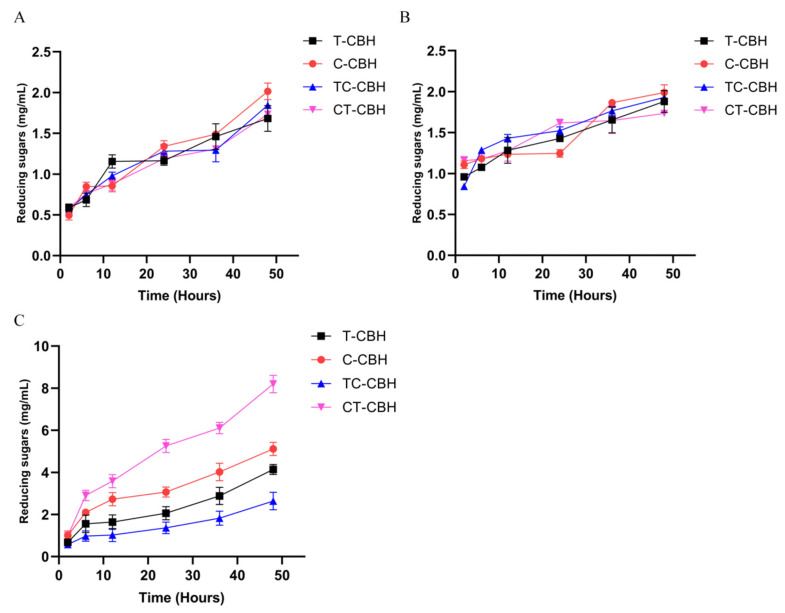
The hydrolytic capacity of four recombinant CBHIs in different biomasses. Determination of the hydrolytic capacity of four recombinant CBHIs on bagasse (**A**), corn straw (**B**), and xylan (**C**). The reducing sugar of the hydrolysate was determined using the DNS method. The values show the mean of three biological replicates and the error bar indicates the standard deviation. T-CBH: *T. reesei* CBHI; C-CBH: *C. thermophilum* CBHI; TC-CBH: chimeric construct with the CBM of *T. reesei* CBHI changed to that of *C. thermophilum*; CT-CBH: chimeric construct with the CBM of *C. thermophilum* CBHI replaced by that of *T. reesei*.

The catalyzing abilities of the four CBHIs on hydrolyzing different biomasses were further compared. The released sugars from bagasse and corn stover, as estimated by glucose equivalents, were very similar for all four CBHIs (Figure 3A,B). This finding aligns closely with previous reports, despite the use of different enzymes [20]. The non-significant difference may arise from the very complex composition of the selected biomasses. Note, even for Avicel and filter paper, the two crystalline cellulose with different structures, the domain swapping had very different effects (compare the specific activities of C-CBH and CT-CBH for Avicel and filter paper, Table 2). Compared to bagasse and corn stover, xylan is a much simpler substrate. Cellobiohydrolases are well-known to have promiscuous activity on this substrate [21]. The abilities of the four CBHIs to degrade xylan were much different (Figure 3C). At the end of the reaction, the released sugars from xylan by T-CBH, C-CBH, TC-CBH, and CT-CBH were 4.1 mg/mL, 5.1 mg/mL, 2.6 mg/mL, and 8.2 mg/mL, respectively (Figure 3C). Therefore, while changing CBM decreased the ability of T-CBH to degrade xylan, changing CBM improved the ability of C-CBH to hydrolyze xylan. CT-CBH demonstrated the highest catalytic activity on xylan compared to the other three CBHIs, which is consistent with the trend in enzyme activity. This suggested that the CBM domains of CBHI have very important but different effects on the hydrolysis of different biomasses.

### 2.6. Binding of Recombinant CBHIs on Crystalline Cellulose

**Figure 4:**
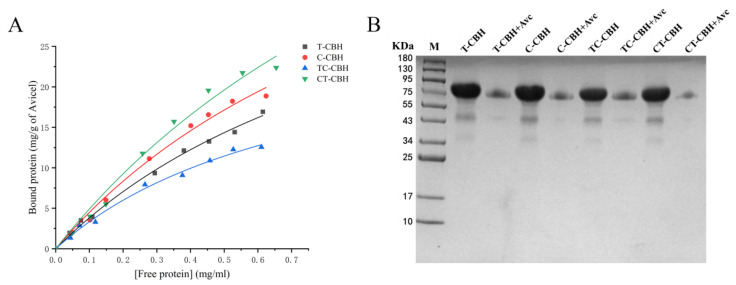
Binding analysis of four recombinant CBHIs to Avicel. (**A**) Quantitative studies of the binding of the four recombinant CBHIs to Avicel. Avicel (20 mg) was mixed with various indicated concentrations of proteins to measure the binding activities. The graph depicts the binding isotherms between bound proteins and free proteins. The values show the mean of three biological replicates. (**B**) Qualitative studies of the binding of the four recombinant CBHIs to Avicel. Avicel PH-101 (Avc) was incubated with 1.5 µM protein. Lane T-CBH, Lane C-CBH, Lane TC-CBH, and Lane CT-CBH represent 1.5 µM protein incubated in the citrate buffer (50 mM, pH 4.8), but without Avicel. The supernatants after incubation of proteins with Avicel were loaded on SDS-PAGE as CBHI + Avc. T-CBH: *T. reesei* CBHI; C-CBH: *C. thermophilum* CBHI; TC-CBH: chimeric construct with the CBM of *T. reesei* CBHI changed to that of *C. thermophilum*; CT-CBH: chimeric construct with the CBM of *C. thermophilum* CBHI replaced by that of *T. reesei*.

Next, the binding abilities of the four CBHIs on the crystalline cellulose (Avicel) were measured. As can be seen from the binding isotherms shown in Figure 4, the binding abilities of the recombinant CBHIs for Avicel varied a lot. For T-CBH, when its CBM was changed, the *K_p_* increased from 0.9 to 1.3 mg/mL and *q_max_* decreased from 45.9 to 29.0 mg protein/g Avicel (for TC-CBH) (Table 3). In contrast, changing the CBM improved the binding of C-CBH to Avicel, with the *K_p_* decreased from 0.8 to 0.7 mg/mL and *q_max_* increased from 57.6 to 77.0 mg protein/g Avicel. These results clearly indicated that the binding abilities of CBHIs to crystalline cellulose could be altered, although the effects were much different. This aligns with the previous research emphasizing the role of CBMs in degrading crystalline cellulose, and the interactions of CBMs with recalcitrant components in heterogeneous substrates can influence hydrolysis at different stages [15].

## 3. Materials and Methods

### 3.1. Microbial Strains and Growth Conditions

*Escherichia coli* DH5α was used for recombinant plasmid construction. The *T. reesei* Tu-6∆*ku70* strain, utilized as the host for the expression of recombinant CBHIs, was kept in this laboratory. *T. reesei* QM6a was ordered from the American Type Culture Collection (Manassas, VA, USA). The recombinant *T. reesei* strains used to produce CBHI proteins (Tr-pT*cbh1*, Tr-pC*cbh1*, Tr-pTC*cbh1*, Tr-pCT*cbh1*) were constructed in this study and will be described below. The *C. thermophilum* (CGMCC 3.17990) was from the CGMCC (China General Microbiological Culture Collection Center, Beijing, China). All *T. reesei* strains were grown for 7 days at 28 °C on potato dextrose agar (PDA) plates or PDA supplemented with 5 mM uridine when necessary. The minimal medium (MM) served as a selective medium to screen *T. reesei* transformants [22].

### 3.2. Transforamtion of T. reesei

*T. reesei* was transformed by electroporation using a protocol derived from X. Jiang et al. [6], with modifications to enhance transformation efficiency. Fresh conidia were harvested from PDA plates, washed three times with ice-cold 1.1 M sorbitol, and resuspended at a concentration of 10^8^ conidia/mL. A mixture of 2–3 µg of DNA in 10 µL was combined with 90 µL of the conidial suspension, placed in a pre-chilled electroporation cassette, and subjected to electroporation using the Gene Pulser Xcell system (Bio-Rad, Hercules, CA, USA) with the following parameters: 1.6 kV, 600 Ω, and 25 µF. After electroporation, 900 µL of ice-cold sorbitol was added, and the mixture was transferred to 10 mL of YPD (1% yeast extract, 2% peptone, 1% dextrose) for 12 h at 28 °C. The mixture was then centrifuged at 4000× *g* for 5 min, resuspended in 1 mL of YPD, washed twice with MM, and mixed with 15 mL of MM containing 2% agar before being spread onto two MM plates. The plates were incubated at 28 °C for 5 days.

### 3.3. Construction of the Recombinant T. reesei Strains

The *cbh1* promoter upstream sequence, the *pyr4* expression cassette, the *cDNA1* promoter, and the downstream of the *cbh1* promoter (containing *T. reesei cbh1* gene) were amplified from the genome of QM6a by PCR. The plasmid pT*cbh1* was constructed by ligating the above fragments to the pBluescript SK (+) plasmid backbone by Gibson assembly (Figure 1B). The *C. thermophilum cbh1* (Accession No: AM711862.1) gene was amplified from the genome of *C. thermophilum* (CGMCC 3.17990). Plasmids pC*cbh1*, pTC*cbh1*, and pCT*cbh1* were constructed as same as plasmid pT*cbh1*. The schematic representation of the recombinant plasmid is shown in Figure S1. The specific primers used are listed in Table S1.

Plasmids pT*cbh1*, pC*cbh1*, pTC*cbh1*, and pCT*cbh1* were transformed into the *T. reesei* Tu-6∆*ku70* strain and inserted into the *T. reesei cbh1* site to construct the *T. reesei* strains Tr-pT*cbh1*, Tr-pC*cbh1*, Tr-pTC*cbh1*, and Tr-pCT*cbh1*. All strains were isolated using minimal media agar plates, and positive colonies were identified through diagnostic PCR. Genotyping of the construction of recombinant CBHI homokaryotic strains is shown in Figures S2 and S3.

### 3.4. Expression and Purification of the CBHIs

For expression of recombinant proteins, the *T. reesei* conidia were inoculated into 50 mL of liquid minimal medium with 2% glucose as the carbon source (MM + 2% glucose) at 10^6^ conidia/mL and then incubated for 48 h at 28 °C on a rotary shaker at 220 rpm.

To purify CBHIs, the crude enzymes were filtered through a 0.22 μm filter membrane to remove any debris and impurity and were concentrated by ultrafiltration using 30 kDa Amicon Ultra-15 centrifugal filter units (Millipore, Billerica, MA, USA). The buffer of the concentrated protein was changed to a TBS buffer (containing 50 mM Tris-HCl, 150 mM NaCl, pH 7.5).

### 3.5. Assay of the Enzyme Activities

The method for measuring cellobiohydrolase (CBH) activity was described by Bailey and Tähtiharju et al. with slight modification [23]. Briefly, 10 µL of enzymes and 40 µL of 1 mM of 4-methylumbelliferyl-β-D-cellobiose (MUC, Sigma, St. Louis, MO, USA) dissolved in a 50 mM citric acid sodium citrate buffer (pH 4.8) were mixed and incubated at 50 °C for 3 min. The reaction was terminated by adding 100 µL of 1 M Na_2_CO_3_. The fluorescence was measured at 445 nm with excitation at 365 nm. One unit of CBH activity was defined as the amount of enzyme required to release 1 μmol of methylumbelliferone in one minute.

To determine the specific activity of recombinant CBHIs on different cellulose, Avicel, phosphoric acid swollen cellulose (PASC), and filter paper were used as substrates. The release of reducing sugars was measured using the 2,5-dinitrosalicylic acid (DNS) method [24]. Avicel PH-101, a nearly pure cellulose, was purchased from Aladdin (Shanghai, China). The PASC production method was slightly modified from that described by Jian Du et al. [15]. These methods of determining the specific activity were described by Zhuolin Yi et al. with slight modifications [25]. In brief, 200 µL of enzymes were incubated with 800 µL 1% (*w*/*v*) of Avicel or 800 µL 1% (*w*/*v*) of PASC in a 50 mM citric acid sodium citrate buffer (pH 4.8) incubated for 1 h at 50 °C. When using filter paper as a substrate, 0.5 mL of enzymes were incubated with Whatman No. 1 filter paper (6 cm × 0.8 cm, Whatman, Maidstone, UK) in 1.5 mL of 50 mM citric acid sodium citrate buffer (pH 4.8) for 1 h at 50 °C. A control was prepared by incubating the substrates without any enzymes under the same reaction conditions. One unit of the activity is defined as the amount of enzymes required to produce one μmol of reducing sugar per minute from Avicel, PASC, or filter paper at 50 °C.

The kinetic parameters of the recombinant CBHIs were determined in the same citrate buffer. The *K_M_* and *k*_cat_ values were estimated by fitting the data to the Michaelis-Menten equation using a nonlinear regression method (GraphPad Prism v5.01 Software, San Diego, CA, USA). These methods were executed as outlined above.

### 3.6. Effects of the pH and Temperature on the Activity of CBHIs

The optimal pHs of the enzymes were determined by measuring the enzyme activities at 50 °C in buffers with different pHs (50 mM Glycine-HCl, pH 2.0–3.0; 50 mM Citric buffer, pH 3.0–6.0, 200 mM phosphate buffer, pH 6.0–8.0, and 50 mM Glycine-NaOH, pH 9.0–10.0). The CBH activity was performed as described above in Section 3.5, “Assay of the enzyme activities”.

The pH stability was assayed by incubating each of the enzymes in the above-mentioned buffers with the pHs ranging from pH 3.0–10.0 for 1 h at room temperature. Then the samples were diluted 10-fold in 50 mM citric buffer, pH 4.8 before measuring the residual activity.

The optimum temperatures of recombinant CBHIs were determined between 30 and 80 °C in the citric buffer (50 mM, pH 4.8). Thermal stability was investigated by incubating the enzyme at different temperatures ranging from 30 to 80 °C for 1 h in 50 mM citric buffer, pH 4.8. The remaining activities were measured under standard conditions.

### 3.7. Hydrolysis of Biomasses

The method was described by Yong Xue et al. with slight modifications [26]. The reaction system included 0.6 g substrate (dry weight), 3 mL crude enzymes, and 27 mL citrate buffer (50 mM, pH 4.8) and was incubated at 50 °C with shaking at 150 rpm. Samples were collected at 6 h or 12 h intervals, and boiled for 5 min to terminate the reaction. The reducing sugar of the hydrolysate was determined using the DNS method [24].

### 3.8. Binding of Insoluble Cellulose

The method for binding insoluble cellulose was described by S. Yoshida et al. with slight modifications [27]. The protein concentration of the enzyme solution was diluted to 0.1 mg/mL–1.8 mg/mL with the citrate buffer (50 mM, pH 4.8). The qualitative binding of proteins to Avicel was assessed as follows: The reaction system included 20 mg Avicel, 200 µL of enzymes with indicated concentrations, and 800 µL citrate buffer (50 mM, pH 4.8) and was incubated at 50 °C. The supernatant was used for the quantification of the unbound (free) protein. Total protein was measured after incubating protein without Avicel under the same conditions. Bound protein was calculated by subtracting the free protein from the total protein. Depletion binding isotherms were derived from the binding of proteins to substrates at different concentrations and were used to quantitatively assess the binding capacity of proteins on Avicel. Protein concentration was determined by the Bradford method by following the instructions (Bio-Rad Protein Assay, Bio-Rad, Hercules, CA, USA).

For the determination of the binding constant between the protein and cellulose, the Michaelis/Langmuir equation was applied. The equation is as follows: *q*_ad_/*q* = *K_p_* × *q_max_*/(1 + *K_p_* × *q*), where *q*_ad_ is the amount of bound protein (mg of proteins per g of cellulose), *q* is the free protein in buffer (mg/mL), *K_p_* is the dissociation constant (mg/mL), and *q_max_* is the maximum amount of bound protein to ligand [28]. The statistical software OriginPro 2022 (OriginLab, Northampton, MA, USA) was utilized for the calculation of the binding parameters.

### 3.9. Statistical Significance Tests

For all the experiments, three biologically replicated strains and three technical replicates for each strain were set for statistical analysis. Statistical significance was determined by ordinary one-way ANOVA and multiple comparison approach by using the GraphPad Prism v5.01 Software (San Diego, CA, USA).

## 4. Conclusions

The replacement of the carbohydrate-binding module (CBM) in *C. thermophilum* CBHI with that from *T. reesei* CBHI resulted in a significant enhancement of recombinant expression levels (Figure 3C). Additionally, domain swapping between the two CBHIs influenced various catalytic properties, particularly their activities on diverse substrates, including the artificial substrate MUC, crystalline cellulose (Avicel), and the promiscuous substrate xylan, with varying effects. Notably, the specific activity of the CT-CBH variant on MUC was 734.5 U/µM, which is 2.6-fold higher than that of *T. reesei* CBHI (T-CBH) at 282.5 U/µM (Table 1). Furthermore, CT-CBH exhibited superior catalytic efficiencies for MUC and PASC, with *k_cat_*/*K_M_* values of 168.9 (1/min)/(mmol/L) for MUC and 6 mg/mL for PASC, compared to T-CBH’s values of 61.6 (1/min)/(mmol/L) for MUC and 3.7 mg/mL for PASC (Table 2). CT-CBH also demonstrated exceptional hydrolytic performance on filter paper, achieving a maximum degradation rate of 11.7 µM glucose equivalents/min/µM enzyme, compared to T-CBH 9.9 µM glucose equivalents/min/µM (Table 2). Additionally, CT-CBH demonstrated a significantly higher hydrolysis capacity on xylan (Figure 3C) and an improved binding affinity to Avicel compared to T-CBH (Table 3, Figure 4A).

The findings demonstrate that exchanging carbohydrate-binding modules (CBMs) significantly influences enzyme binding capacity and catalytic efficiency. Domain swapping between species did not negatively impact pH and temperature stability or solubility, indicating the robustness of these engineered variants. Subsequent analyses employed AlphaFold, which confirmed this observation (Figure S4). Future research will focus on elucidating the structural basis of these effects through molecular dynamics simulations and X-ray crystallography to clarify the interaction networks between CBM and catalytic domain (CD) modules. Additionally, substrate testing will be expanded to include diverse biomass materials. The enhanced activity of CT-CBH on xylan and other substrates suggests its potential as a valuable component in enzyme blends for improved industrial applications. These results highlight the utility of cross-species domain engineering as a promising strategy for optimizing cellulases.

## Figures and Tables

**Table 1:** Kinetic analysis of the CBHIs on MUC.

Enzyme	Specific Activity (U/µM)	*K_M_* (mmol/L)	*k_cat_* (min^−1^)	*k_cat_*/*K_M_* (1/min)/(mmol/L)
T-CBH	282.5 ± 21.12 ^ns^	1.3	77.5	61.6
C-CBH	320.2 ± 35.23 ^ns^	1.1	93.7	88.5
TC-CBH	301.3 ± 20.68 ^ns^	1.2	172.5	141.8
CT-CBH	734.5 ± 18.83 ****	0.7	115.1	168.9

The reactions were performed in a 50 mM citric acid sodium citrate buffer (pH4.8) at 50 °C. T-CBH: *T. reesei* CBHI; C-CBH: *C. thermophilum* CBHI; TC-CBH: chimeric construct with the CBM of *T. reesei* CBHI changed to that of *C. thermophilum*; CT-CBH: chimeric construct with the CBM of *C. thermophilum* CBHI replaced by that of *T. reesei*. The values show the mean of three biological replicates. Data are shown as means ± standard errors. ****: *p* < 0.0001, indicating a statistically significant difference; ^ns^: no significant difference (*p* > 0.05).

**Table 2:** Kinetic parameters of CBHIs of different cellulosic substrates.

Enzyme	PASC			Avicel *^a^* (µM Glucose Equivalents/min/µM Enzyme)			Filter Paper *^b^* (µM Glucose Equivalents/min/µM Enzyme)
	*k_cat_* (s^−1^)	*K_m_* (mg/mL)	*k_cat_* (s^−1^)/*K_m_* (mg/mL)				
T-CBH	2.0 × 10^−1^	5.6 × 10^−2^	3.7	3.8 ± 0.4	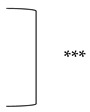		9.9 ± 1.9 ^ns^
C-CBH	3.3 × 10^−1^	7.3 × 10^−2^	4.5	3.1 ± 0.4		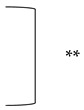	10.0 ± 1.9 ^ns^
TC-CBH	3.2 × 10^−1^	1.2 × 10^−1^	2.7	2.0 ± 0.1			7.5 ± 2.2 ^ns^
CT-CBH	2.1 × 10^−1^	3.5 × 10^−2^	6.0	2.0 ± 0.2			11.7 ± 2.4 ^ns^

The reactions were performed in a 50 mM citric acid sodium citrate buffer (pH 4.8) at 50 °C. PASC: phosphoric acid swollen cellulose. T-CBH: *T. reesei* CBHI; C-CBH: *C. thermophilum* CBHI; TC-CBH: chimeric construct with the CBM of *T. reesei* CBHI changed to that of *C. thermophilum*; CT-CBH: chimeric construct with the CBM of *C. thermophilum* CBHI replaced by that of *T. reesei*. The values show the mean of three biological replicates. Data are shown as means ± standard errors. *^a^* TC-CBH vs. T-CBH: *** *p* < 0.001, indicating a statistically significant difference between the two groups; CT-CBH vs. C-CBH: ** *p* < 0.01, indicating a statistically significant difference between the two groups; *^b^* ^ns^: no significant difference (*p* > 0.05).

**Table 3:** The binding parameters of recombinant CBHIs for Avicel.

Protein	*K_P_* (mg/mL)	*q_max_* (mg Protein/g Avicel)
T-CBH	0.9 ± 0.2 ^ns^	45.9 ± 7.9
C-CBH	0.8 ± 0.3 ^ns^	57.6 ± 13.4
TC-CBH	1.3 ± 0.3 ^ns^	29.1 ± 4.6
CT-CBH	0.7 ± 0.3 ^ns^	77.0 ± 26.9

*K_P_* is the dissociation constant (mg/mL), and *q_max_* is the maximum amount of bound protein to the ligand. T-CBH: *T. reesei* CBHI; C-CBH: *C. thermophilum* CBHI; TC-CBH: chimeric construct with the CBM of *T. reesei* CBHI changed to that of *C. thermophilum*; CT-CBH: chimeric construct with the CBM of *C. thermophilum* CBHI replaced by that of *T. reesei*. The values show the mean of three biological replicates. Data are shown as means ± standard errors. ^ns^: no significant difference (*p* > 0.05).

## Data Availability

Data are contained within the article and Supplementary Materials.

## Supplementary Materials

The supporting information can be downloaded at: https://www.mdpi.com/article/10.3390/ijms26094024/s1.
