# Supplementary material for: Catalytic Efficiency Improvement in Cellobiohydrolase I by Cross-Species Domain Exchange Engineering
Source: Int J Mol Sci. 2025 Apr 24;26(9):4024. doi: 10.3390/ijms26094024 (PMC12072009; doi:10.3390/ijms26094024)
Supplement: Supplementary file 1 [file ijms-26-04024-s001.zip › ijms-3552207-supplementary.pdf]

Highlights

- Domain swapping resulted in the creation of a superior cellobiohydrolase I (CT-CBH).
- The structure of CT-CBH specifically contributed to its high expression levels.
- The specific structure of CT-CBH contributed to its enhanced specific enzyme activity.

Supplementary data

1. Schematic representation of the recombinant plasmid

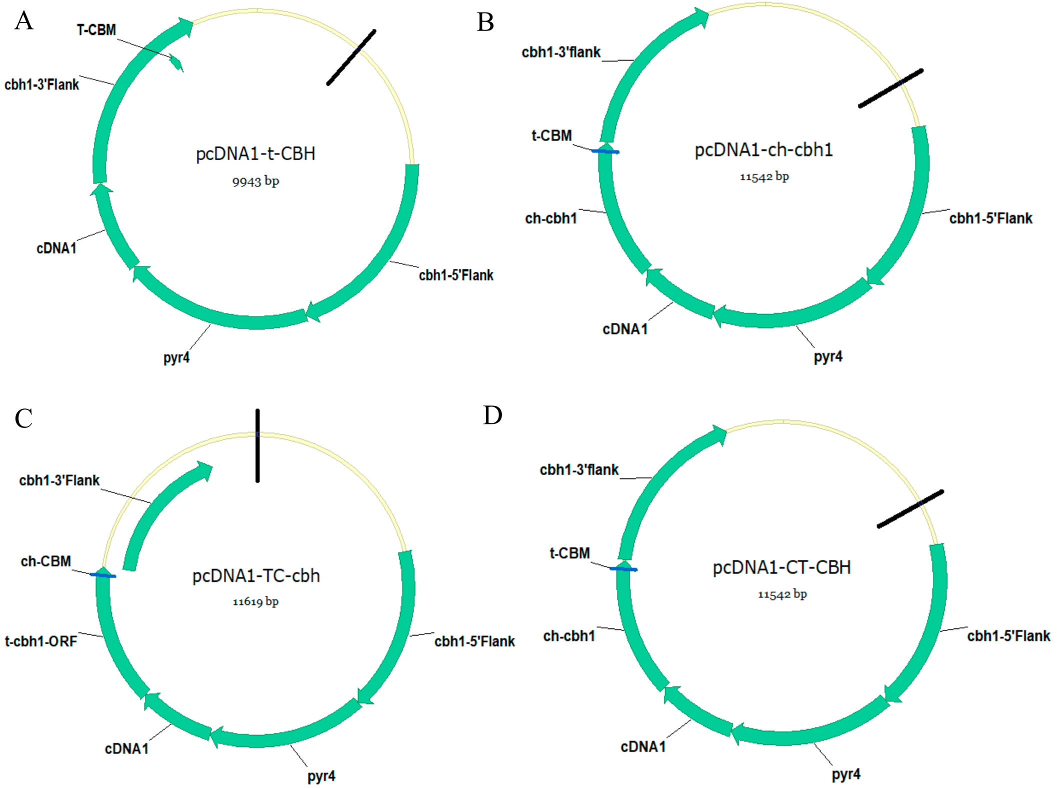

Figure S1. Schematic diagram of vector construction of four recombinant CBHIs.
(A) pT*cbh1*; (B) pC*cbh1*; (C) pTC*cbh1*; (B) pCT*cbh1*

2. ***cDNA sequence of the target gene*** **(Black font represents the catalytic domain, red font represents the linker, and blue font represents the carbohydrate-binding module)**

T-CBH:

atgtatcggaagttggccgtcatctcggccttcttggccacagctcgtgctcagtcggcc
tgcactctccaatcggagactcacccgcctctgacatggcagaaatgctcgtctggtggc
acgtgcactcaacagacaggctccgtggtcatcgacgccaactggcgctggactcacgct
acgaacagcagcacgaactgctacgatggcaacacttggagctcgaccctatgtcctgac
aacgagacctgcgcgaagaactgctgtctggacggtgccgcctacgcgtccacgtacgga
gttaccacgagcggtaacagcctctccattggctttgtcacccagtctgcgcagaagaac
gttggcgctcgcctttaccttatggcgagcgacacgacctaccaggaattcaccctgctt
ggcaacgagttctctttcgatgttgatgtttcgcagctgccgtgcggcttgaacggagct
ctctacttcgtgtccatggacgcggatggtggcgtgagcaagtatcccaccaacaccgct
ggcgccaagtacggcacggggtactgtgacagccagtgtccccgcgatctgaagttcatc
aatggccaggccaacgttgagggctgggagccgtcatccaacaacgcgaacacgggcatt
ggaggacacggaagctgctgctctgagatggatatctgggaggccaactccatctccgag
gctcttacccccccacccttgcacgactgtcggccaggagatctgcgagggtgatgggtgc
ggcggaacttactccgataacagatatggcggcacttgcgatcccgatggctgcgactgg
aacccataccgcctgggcaacaccagcttctacggccctggctcaagctttaccctcgat
accaccaagaaattgaccgttgtcacccagttcgagacgtcgggtgccatcaaccgatac
tatgtccagaatggcgtcactttccagcagcccaacgccgagcttggtagttactctggc
aacgagctcaacgatgattactgcacagctgaggaggcagaattcggcggatcctctttc
tcagacaagggcggcctgactcagttcaagaaggctacctctggcggcatggttctggtc
atgagtctgtgggatgattactacgccaacatgctgtggctggactccacctacccgaca
aacgagacctcctccacacccggtgccgtgcgcggaagctgctccaccagctccggtgtc
cctgctcaggtcgaatctcagtctcccaacgccaaggtcaccttctccaacatcaagttc
ggacccattggcagcaccggcaaccctagcggcggcaaccctcccggcggaaacccgcct
ggcaccaccaccacccgccgcccagccactaccactggaagctctcccggacctacccag
tctcactacggccagtgcggcggtattggctacagcggccccacggtctgcgccagcggc
acaacttgccaggtcctgaacccttactactctcagtgcctgtaa

C-CBH:

atgatgtataagaagttcgccgctctcgccgccctcgtggctggcgcctccgcccagcag
gcttgctccctcaccgctgagaaccaccctagcctcacctggaagcgctgcacctctggc
ggcagctgctcgaccgtgaacggcgccgtcaccatcgatgccaactggcgctggactcac
accgtctccggctcgaccaactgctacaccggcaaccagtgggatacctccctctgcact
gatggcaagagctgcgcccagacctgctgcgtcgatggcgctgactactcttcgacctat
ggtatcaccaccagcggtgactccctgaacctcaagttcgtcaccaagcaccagtacggc
accaacgtcggctcccgtgtctatctgatggagaacgacaccaagtaccagatgttcgag
ctcctcggcaacgagttcaccttcgatgtcgatgtctccaacctgggctgcggtctcaac
ggcgccctctacttcgtttccatggatgctgatggtggcatgagcaaatactctggcaac
aaggctggcgccaagtacggtaccggctactgcgatgctcagtgcccgcgcgacctcaag
ttcatcaacggcgaggccaacgttgggaactggacccccctcgaccaacgatgccaacgcc
ggcttcggccgctatggcagctgctgctctgagatggatgtctgggaggccaacaacatg
gctactgccttcactcctcacccttgcaccaccgttggccagagccgctgcgaggccgac

acctgcggtggcacctacagctctgaccgctatgctggtgtttgcgaccctgatggctgc
gacttcaacgcctaccgccaaggcgacaagaccttctacggcaagggcatgactgtcgac
accaacaagaagatgaccgtcgtcacccagttccacaagaactcggctggcgtcctcagc
gagatcaagcgcttctacgtccaggacggcaagatcattgccaacgctgagtccaagatc
cccggcaaccccggaaactccattacccaggagtattgcgatgcccagaaggtcgccttc
agtaacaccgatgacttcaaccgcaagggcggtatggctcagatgagcaaggccctcgca
ggccccatggtcctggtcatgtccgtctgggatgaccactacgccaacatgctctggctc
gactcgacctaccccatcgaccaggccggcgcccccggcgccgagcgcggtgcttgcccg
accacctccggtgtccctgccgagatcgaggcccaggtccccaacagcaacgtcatcttc
tccaacatccgtttcggccccatcggctcgaccgtccctggccttgacggcagcaacccc
ggcaacccgaccaccaccgtcgttcctcccgcttctacctccacctcccgtccgaccagc
agcactagctctcccgtttcgaccccgactggccagcccggcggctgcaccacccagaag
tggggccagtgcggcggtatcggctacaccggctgcactaactgcgttgctggcaccacc
tgcactcagctcaacccctggtacagccag

TC-CBH:

atgtatcggaagttggccgtcatctcggccttcttggccacagctcgtgctcagtcggcc
tgcactctccaatcggagactcacccgcctctgacatggcagaaatgctcgtctggtggc
acgtgcactcaacagacaggctccgtggtcatcgacgccaactggcgctggactcacgct
acgaacagcagcacgaactgctacgatggcaacacttggagctcgaccctatgtcctgac
aacgagacctgcgcgaagaactgctgtctggacggtgccgcctacgcgtccacgtacgga
gttaccacgagcggtaacagcctctccattggctttgtcacccagtctgcgcagaagaac
gttggcgctcgcctttaccttatggcgagcgacacgacctaccaggaattcaccctgctt
ggcaacgagttctctttcgatgttgatgtttcgcagctgccgtgcggcttgaacggagct
ctctacttcgtgtccatggacgcggatggtggcgtgagcaagtatcccaccaacaccgct
ggcgccaagtacggcacggggtactgtgacagccagtgtccccgcgatctgaagttcatc
aatggccaggccaacgttgagggctgggagccgtcatccaacaacgcgaacacgggcatt
ggaggacacggaagctgctgctctgagatggatatctgggaggccaactccatctccgag
gctcttacccccacccttgcacgactgtcggccaggagatctgcgagggtgatgggtgc
ggcggaacttactccgataacagatatggcggcacttgcgatcccgatggctgcgactgg
aacccataccgcctgggcaacaccagcttctacggccctggctcaagctttaccctcgat
accaccaagaaattgaccgttgtcacccagttcgagacgtcgggtgccatcaaccgatac
tatgtccagaatggcgtcactttccagcagcccaacgccgagcttggtagttactctggc
aacgagctcaacgatgattactgcacagctgaggaggcagaattcggcggatcctctttc
tcagacaagggcggcctgactcagttcaagaaggctacctctggcggcatggttctggtc
atgagtctgtgggatgattactacgccaacatgctgtggctggactccacctacccgaca
aacgagacctcctccacacccggtgccgtgcgcggaagctgctccaccagctccggtgtc
cctgctcaggtcgaatctcagtctcccaacgccaaggtcaccttctccaacatcaagttc
ggacccattggcagcaccggcaaccctagcggcggcaaccctcccggcggaaacccgcct
ggcaccaccaccacccgccgcccagccactaccactggaagctctcccggacctacccag
tctaagtggggccagtgcggcggtatcggctacaccggctgcactaactgcgttgctggcaccacc
tgcactcagctcaacccctggtacagccag

CT-CBH:

atgatgtataagaagttcgccgctctcgccgccctcgtggctggcgcctccgcccagcag

gcttgctccctcaccgctgagaaccaccctagcctcacctggaagcgctgcacctctggc
ggcagctgctcgaccgtgaacggcgccgtcaccatcgatgccaactggcgctggactcac
accgtctccggctcgaccaactgctacaccggcaaccagtgggatacctccctctgcact
gatggcaagagctgcgcccagacctgctgcgtcgatggcgctgactactcttcgacctat
ggtatcaccaccagcggtgactccctgaacctcaagttcgtcaccaagcaccagtacggc
accaacgtcggctcccgtgtctatctgatggagaacgacaccaagtaccagatgttcgag
ctcctcggcaacgagttcaccttcgatgtcgatgtctccaacctgggctgcggtctcaac
ggcgccctctacttcgtttccatggatgctgatggtggcatgagcaaatactctggcaac
aaggctggcgccaagtacggtaccggctactgcgatgctcagtgcccgcgcgacctcaag
ttcatcaacggcgaggccaacgttgggaactggacccccctcgaccaacgatgccaacgcc
ggcttcggccgctatggcagctgctgctctgagatggatgtctgggaggccaacaacatg
gctactgccttcactcctcacccttgcaccaccgttggccagagccgctgcgaggccgac
acctgcggtggcacctacagctctgaccgctatgctggtgtttgcgaccctgatggctgc
gacttcaacgcctaccgccaaggcgacaagaccttctacggcaagggcatgactgtcgac
accaacaagaagatgaccgtcgtcacccagttccacaagaactcggctggcgtcctcagc
gagatcaagcgcttctacgtccaggacggcaagatcattgccaacgctgagtccaagatc
cccggcaaccccggaaactccattacccaggagtattgcgatgcccagaaggtcgccttc
agtaacaccgatgacttcaaccgcaagggcggtatggctcagatgagcaaggccctcgca
ggccccatggtcctggtcatgtccgtctgggatgaccactacgccaacatgctctggctc
gactcgacctaccccatcgaccaggccggcgcccccggcgccgagcgcggtgcttgcccg
accacctccggtgtccctgccgagatcgaggcccaggtccccaacagcaacgtcatcttc
tccaacatccgtttcggccccatcggctcgaccgtccctggccttgacggcagcaacccc
ggcaacccgaccaccaccgtcgttcctcccgcttctacctccacctcccgtccgaccagc
Agcactagctctcccgtttcgaccccgactggccagcccggcggctgcaccacccag
cactacggccagtgcggcggtattggctacagcggccccacggtctgcgccagcggc
acaacttgccaggtcctgaacccttactactctcagtgcctgtaa

3. Genotyping of the construction of recombinant CBHI homokaryotic strains

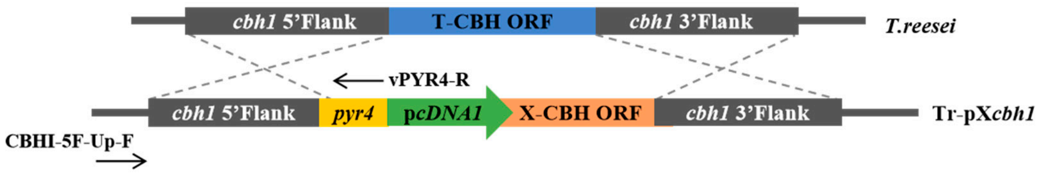

Figure S2. Schematic diagram of vector construction of four recombinant CBHIs.
The vector consists of the upstream sequence of the *cbh1* promoter, the *pyr4* expression cassette, the *cDNA1* promoter, the X-CBH ORF, the downstream sequence of the *cbh1* promoter, and the pBluescript SK(+) plasmid backbone. X represents T, C, TC, and CT; Tr-pX*cbh1* represents Tr-pT*cbh1*, Tr-pC*cbh1*, Tr-pTC*cbh1*, and Tr-pCT*cbh1*, respectively. T-CBH: *T. reesei* CBHI; C-CBH: *C. thermophilum* CBHI; TC-CBH: chimeric construct with the CBM of *T. reesei* CBHI changed to that of *C. thermophilum*; CT-CBH: chimeric construct with the CBM of *C. thermophilum* CBHI replaced by that of *T. reesei*.

All recombinant strains were isolated using agar plates and positive colonies were identified by PCR. Transformants were amplified using external validation primers

CBHI-5F-Up-F and vPYR4-R, which showed a band of 2439 bp in both cases, whereas the corresponding band was not detected in the negative control strain (Tu6), suggesting that the target fragment had been successfully targeted for insertion into the *cbh1* locus of *T. reesei* (Figure S2). Primers Pcbh1-1F/1R, targeting the *cbh1* locus, revealed no amplification in transformants, while a 1365 bp product was detected in the negative control strain Tu6 (Figure S3), demonstrating complete replacement of the endogenous *cbh1* gene and successful generation of four recombinant CBHI homokaryotic strains.

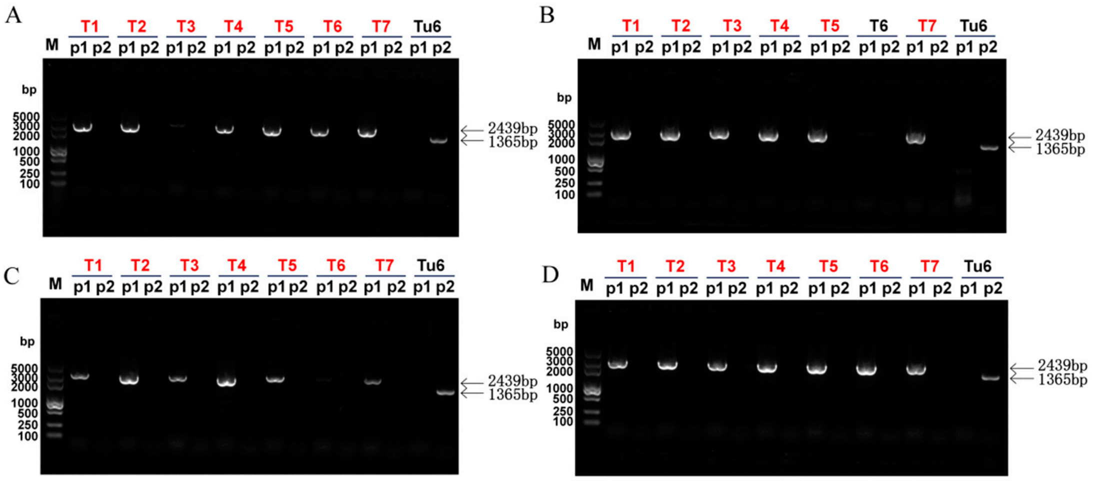

Figure S3. PCR verification of recombinant CBHI homokaryotic strains.
Figures (A), (B), (C) and (D) show the results of the homokaryotic strains screening of Tr-pT*cbh1*, Tr-pC*cbh1*, Tr-pTC*cbh1* and Tr-pCT*cbh1* of *T. reesei* respectively. Transformants amplified with primers CBHI-5F-Up-F/vPYR4-R showed a 2439 bp band, which was absent in the negative control (Tu6), confirming successful insertion into the *cbh1* locus. Using primers Pcbh1-1F/1R, transformants showed no bands, while a 1365 bp band was detected in the negative control, indicating the successful construction of recombinant CBHI homokaryotic strains. The red markings indicate the correctly screened recombinant CBHI homokaryotic strains.

4. Alphafold prediction and analysis of recombinant CBHI

Research has shown that the structure of CBHI comprises three key functional domains: the N-terminal family 7 glycoside hydrolase CD, the C-terminal CBM, and a flexible linker peptide connecting the two. This study employed AlphaFold for protein structure predictions of recombinant CBHI, analyzing the predicted structures of recombinant CBHI, as illustrated in Figure S4A. The results confirm the typical three-domain features of CBHI, and reveal the structural basis for its catalytic activity

and substrate binding.

To compare the structural differences before and after the replacement of CBM, the impact of the CBM on the CBHI structure was investigated by using AlphaFold predictions. The analysis included comparisons of the structures before and after CBM replacement (T-CBH vs. TC-CBH, RMSD = 0.343; C-CBH vs. CT-CBH, RMSD = 0.354, Figure S4B). The results indicate that replacing the CBM increases overall structural differences (T-CBH vs. C-CBH, RMSD = 0.270; the differences increase after CBM replacement,Figure S4B), likely affecting function. This expectation aligns with our enzyme activity experiments and other results.

Further RMSD analysis of individual domains revealed a CD RMSD of 0.339 (Figure S4C), indicating strong conservation, while the linker showed an RMSD of 1.135 (Figure S4C), reflecting significant differences that may indicate adaptive functional adjustments. The CBM RMSD was only 0.195 (Figure S4C), suggesting relative conservation of its structure. This analysis supports understanding the structure-function relationships of different CBHI proteins and informs future functional validation experiments.

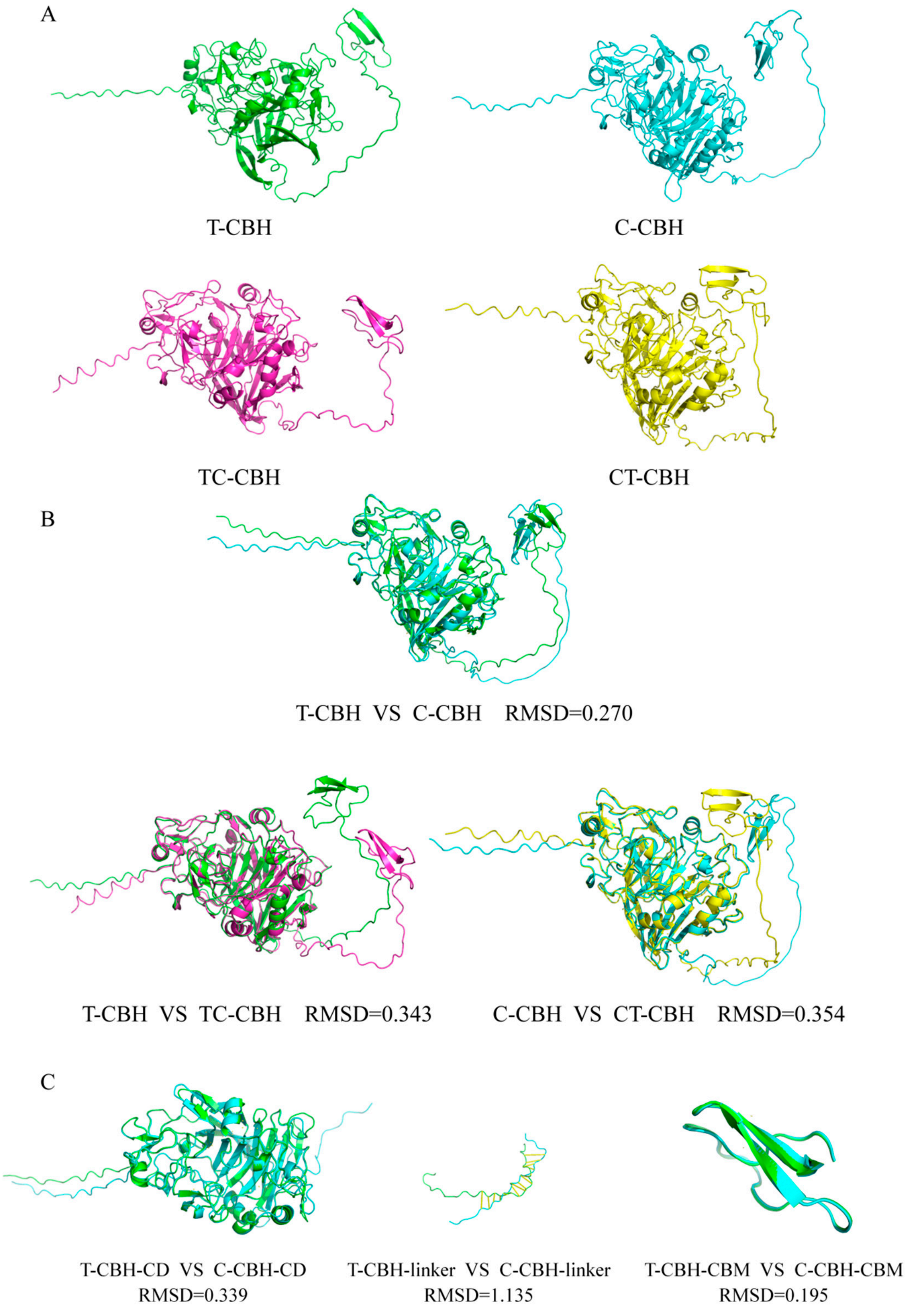

Figure. S4 Predicted protein structure of recombinant CBHI (A), comparison of structural differences between recombinant CBHIs (B), and structural comparison between individual domains (C).

T-CBH: *T. reesei* CBHI; C-CBH: *C. thermophilum* CBHI; TC-CBH: chimeric construct with the CBM of *T. reesei* CBHI changed to that of *C. thermophilum*; CT-CBH: chimeric construct with the CBM of *C. thermophilum* CBHI replaced by that of *T. reesei*.

5. Supplementary Tables

Table S1. Primers used in this study

Primer name	Primer sequence (5' to 3')
**Primers for construction of Tr-pT*cbh1***	
5Tcbh1-F	gggagaccggcagcggccgcatctcgcaacacacacaatg
5Tcbh1-R	ccgatattgcgactttggggaaaatacacagaagacacaa
pyr4-F	ttgtgtcttctgtgtattttccccaaagtcgcaatatcgg
pyr4-R	gctgctaccatcattgtctgcaactgcatccaaaccatcc
cDNA1-F	ggatggtttggatgcagttgcagacaatgatggtagcagc
cDNA1-R	acggccaacttccgatacatgagagaagttgttggattga
Trcbh1-F	tcaatccaacaacttctctcatgtatcggaagttggccgt
Trcbh1-R	ttggagcactgcgaggggccaagagcggcgattctacggg
pln55-F	cccgtagaatcgccgctcttggcccctcgcagtgctccaa
pln55-R	ccgatattgcgactttggggaaaatacacagaagacacaa
**Primers for construction of Tr-pC*cbh1***	
5Tcbh1-F	gggagaccggcagcggccgcatctcgcaacacacacaatg
5Tcbh1-R	ccgatattgcgactttggggaaaatacacagaagacacaa
pyr4-F	ttgtgtcttctgtgtattttccccaaagtcgcaatatcgg
pyr4-R	gctgctaccatcattgtctgcaactgcatccaaaccatcc
cDNA1-F	ggatggtttggatgcagttgcagacaatgatggtagcagc
cDNA1-R	acggccaacttccgatacatgagagaagttgttggattga
pln55-F	cccgtagaatcgccgctcttggcccctcgcagtgctccaa
pln55-R	ccgatattgcgactttggggaaaatacacagaagacacaa
Chcbh1-F	tcaatccaacaacttctctcatgatgtataagaagttcgc
Chcbh1-R	caggctttcgccacggagctttacaggcactggctgtacc
**Primers for construction of Tr-pTC*cbh1***	
pcDNA1-TC-CBH-1F	ggtactctcagtgcctgtaaagctccgtggcgaaagcctg
pcDNA1-TC-CBH-1R	gaaaattggagcactgcgaggggccgtttcgtgcggctgaatcc
pcDNA1-TC-CBH-2F	ggattcagccgcacgaaacggcccctcgcagtgctccaattttc
pcDNA1-TC-CBH-2R	caggctttcgccacggagctttacaggcactgagagtacc
**Primers for construction of Tr-pCT*cbh1***	
pcDNA1-CT-CBH-1F	aggtcctgaacccttactacagccagtgcctgtaaagctc
pcDNA1-ch-CBH1-R	gcgaacttcttatacatcatgagagaagttgttggattga
Chcbh1-F	tcaatccaacaacttctctcatgatgtataagaagttcgc
pcDNA1-CT-CBH-2R	ccgccgcactggccgtagtgctgggtggtgcagccgccgg
CBHI-5F-Up-F	gaggttcgtgataacatggc
vPYR4-R	gtcgggctcgtcccctggtc
Pcbh1-1F	taggatcgaacacactgctg
Pcbh1-1R	tgggctgctggaaagtgacg
